# The Membrane Bound LRR Lipoprotein Slr, and the Cell Wall-Anchored M1 Protein from *Streptococcus pyogenes* Both Interact with Type I Collagen

**DOI:** 10.1371/journal.pone.0020345

**Published:** 2011-05-31

**Authors:** Marta Bober, Matthias Mörgelin, Anders I. Olin, Ulrich von Pawel-Rammingen, Mattias Collin

**Affiliations:** 1 Division of Infection Medicine, Department of Clinical Sciences, Biomedical Center, Lund University, Lund, Sweden; 2 Department of Molecular Biology, Umeå University, Umeå, Sweden; Charité-University Medicine Berlin, Germany

## Abstract

*Streptococcus pyogenes* is an important human pathogen and surface structures allow it to adhere to, colonize and invade the human host. Proteins containing leucine rich repeats (LRR) have been indentified in mammals, viruses, archaea and several bacterial species. The LRRs are often involved in protein-protein interaction, are typically 20–30 amino acids long and the defining feature of the LRR motif is an 11-residue sequence LxxLxLxxNxL (x being any amino acid). The streptococcal leucine rich (Slr) protein is a hypothetical lipoprotein that has been shown to be involved in virulence, but at present no ligands for Slr have been identified. We could establish that Slr is a membrane attached horseshoe shaped lipoprotein by homology modeling, signal peptidase II inhibition, electron microscopy (of bacteria and purified protein) and immunoblotting. Based on our previous knowledge of LRR proteins we hypothesized that Slr could mediate binding to collagen. We could show by surface plasmon resonance that recombinant Slr and purified M1 protein bind with high affinity to collagen I. Isogenic *slr* mutant strain (MB1) and *emm1* mutant strain (MC25) had reduced binding to collagen type I as shown by slot blot and surface plasmon resonance. Electron microscopy using gold labeled Slr showed multiple binding sites to collagen I, both to the monomeric and the fibrillar structure, and most binding occurred in the overlap region of the collagen I fibril. In conclusion, we show that Slr is an abundant membrane bound lipoprotein that is co-expressed on the surface with M1, and that both these proteins are involved in recruiting collagen type I to the bacterial surface. This underlines the importance of *S. pyogenes* interaction with extracellular matrix molecules, especially since both Slr and M1 have been shown to be virulence factors.

## Introduction

*Streptococcus pyogenes* is an important human pathogen. It most commonly causes throat and skin infections, such as pharyngitis and impetigo [1]. It can also cause severe invasive diseases such as sepsis, necrotizing fasciitis and toxic shock syndrome, as well as complications post infection such as rheumatic fever and glomerulonephritis [2]. Surface structures of *S. pyogenes* allow the bacteria to adhere to, colonize and invade mucus membranes and human skin. Some of these structures are M protein, M-like proteins, collagen type I-binding protein (Cpa) and streptococcal fibronectin-binding protein I (SfbI) [3], [4].

Mammalian cells have a variety of ways to detect invading pathogens and to alert the immune system. The best known are the Toll-like receptors (TLR), which are transmembrane receptors containing an extracellular leucine rich repeat (LRR) domain. It is the LRR domain in TLRs that is responsible for recognizing diverse microbial components [5]. More recently indentified receptors involved in bacterial recognition are receptors with a carboxyl-terminal LRR domain, a central nucleotide binding and oligomerization domain (NOD). They are implicated in the cytosolic detection of bacterial components, mediated through the LRR domain [6], [7]. LRR proteins are also involved in protein-protein interactions such as signal transduction, cell adhesion and apoptosis [8], [9]. LRR proteoglycan decorin has been shown to bind to fibrillar collagens. Other examples of LRR proteins are proline arginine-rich end leucine-rich repeat protein (PRELP), chondroadherin (CHAD) and biglycan [10], [11].

LRRs are typically 20–30 amino acids long and the defining feature of the LRR motif is an 11-residue sequence LxxLxLxxNxL (x being any amino acid). The number of LRR repeats ranges from 2 to 45 and they are divided into a conserved and variable segment [12], [13]. The conserved amino-terminal stretch of 9–12 amino acids forms the β-strand and the variable segment is a carboxy-terminal stretch of 10–19 amino acids that varies in length, sequence and structure. The arrangement of the repeats results in a horseshoe-shaped structure with the β-sheet on the concave side and the variable stretches on the convex side [14], [15].

Several pathogenic bacteria, both Gram positive and Gram negative express surface proteins with LRR regions. LRR proteins have also been identified in viruses, archaea and eukaryotes [15], [16]. Internalins of *Listeria monocytogenes* are the most studied bacterial proteins with LRR domains. There are at least 9 proteins in this family and all have been implicated in the invasion of the human cell [17]. The streptococcal leucine rich (Slr) protein is predicted to be a lipoprotein attached to the cell membrane and has been shown to be camouflaged for antibody recognition by the M6 protein [18], [19]. Antibodies against Slr are developed during a *S. pyogenes* infection in humans and an *S. pyogenes* strain lacking Slr had lower resistance against neutrophil phagocytosis *in vitro* and was less virulent in a mouse model of infection [18], [19]. Slr lacks a LPXTG motif, which links Gram-positive proteins to the cell wall; instead a TLIA lipobox is present and acts as a membrane anchoring motif. The Slr protein is similar to virulence proteins of the internalin family of *L. monocytogenes* but there are differences between the proteins. The LRR region in Slr is located in the C-terminal half of the molecule unlike the members of the internalin family in which the LRR region is located in the N-terminal part of the protein. At the end of the N-terminal region there are four histidine triad motifs that are not present in the internalin family of proteins.

Adherence of human pathogens to certain tissue components might reflect preferences for specific sites of infection. Bacterial adhesins that interact with extracellular matrix components such as collagen, fibronectin, fibrinogen and laminin-related polysaccharides have been identified for both Gram-negative and Gram-positive bacteria. Groups A, B, C, D and G streptococci have all been shown to exhibit collagen binding ability [20]. Protein FOG is one of the proteins that recruit collagen type I in group G streptococci [21]. For *S. pyogenes*, adhesion to collagen type IV by surface protein M3 has been reported as well as binding to M-negative strains [2], [22]. Since Slr is a putative surface located lipoprotein containing LRR motifs, we hypothesized that Slr could mediate binding to collagen. In this study we present experimental evidence that Slr is a surface attached lipoprotein containing LRR regions. Furthermore, using electron microscopy of immunogold labeled Slr and other experimental techniques, we were able to show that Slr is a collagen I binding protein.

## Materials and Methods

### Reagents, bacteria and culture conditions

Bacteria and plasmids used in this study are described in Table 1. The *S. pyogenes* strain of serotype M1 (AP1) and mutants MB1 and MC25 were cultured in Todd-Hewitt broth (TH) (Difco, Detroit, DI, USA) or TH broth with 0.2% yeast extract (THY) (Oxoid LTD, Hampshire, UK) at 37°C in 5% CO_2_ atmosphere, including 150 µg/ml kanamycin for growth of the mutants. Collagen I was purchased from Sigma (St.Louis, MO, USA). Antibodies used in this study were goat anti-rabbit IgG-peroxidase labeled antibody (Bio-Rad, CA, USA) and rabbit anti-collagen I/III antibody (Calbiochem, Darmstadt, Germany). The *S. pyogenes* mutant strain MC25 used in this study was constructed as previously described by Collin *et al* [23].

**Table 1:** Bacteria and plasmids used in this study and the presence of *slr* gene in *S. pyogenes* strains.

Strain/plasmid	M type	Genotype	Characteristics	*Slr* gene^1^	Reference or source
Bacteria					
*Streptococcus pyogenes*					
AP1	1	Wild type	M1 serotype	+	WHO Prague collection^2^
MC25	1	*emm1*::Km	M1 lacking mutant	+	[23]
MB1	1	*slr*::Km	Slr lacking mutant	-	This study
SF370	1	*emm1*	M1 serotype	+	[37], [49]
AP4	4	*emm4*	M4 serotype	+	WHO Prague collection
AP6	6	*emm6*	M6 serotype	+	WHO Prague collection
AP12	12	*emm12*	M12 serotype	+	WHO Prague collection
AP15	15	*emm15*	M15 serotype	+	WHO Prague collection
AP23	23	*emm23*	M23 serotype	+	WHO Prague collection
AP46	46	*emm46*	M46 serotype	+	WHO Prague collection
AP47	47	*emm47*	M47 serotype	+	WHO Prague collection
AP53	53	*emm53*	M53 serotype	+	WHO Prague collection
Manfredo	5	*emm5*	M5 serotype	+	[50]
3686-98	1	*emm1*	M1 serotype	+	Center for disease control^3^
3688-98	2	*emm2*	M2 serotype	+	Center for disease control
3671-98	3	*emm3*	M3 serotype	+	Center for disease control
3670-98	4	*emm4*	M4 serotype	+	Center for disease control
3667-98	5	*emm5*	M5 serotype	+	Center for disease control
3693-98	6	*emm6*	M6 serotype	+	Center for disease control
3261-98	9	*emm9*	M9 serotype	+	Center for disease control
3904-98	11	*emm11*	M11 serotype	+	Center for disease control
3664-98	12	*emm12*	M12 serotype	+	Center for disease control
3258-98	18	*emm18*	M18 serotype	+	Center for disease control
3913-98	19	*emm19*	M19 serotype	+	Center for disease control
3858-98	22	*emm22*	M22 serotype	+	Center for disease control
SS-987	24	*emm24*	M24 serotype	+	Center for disease control
3272-98	28	*emm28*	M28 serotype	+	Center for disease control
3274-98	49	*emm49*	M49 serotype	+	Center for disease control
189-98	55	*emm55*	M55 serotype	+	Center for disease control
SS-790	57	*emm57*	M57 serotype	+	Center for disease control
978-97	60	*emm60*	M60 serotype	+	Center for disease control
86-858	12	*emm12*	M12 serotype	+	[51]
KTL3	1	*emm1*	M1 serotype	+	Finnish Institute for Health
KTL9	28	*emm28*	M28 serotype	+	Finnish Institute for Health
*Escherichia coli*					
TOP10		*recA*1 *endA*1 *hsdRMS*	Cloning strain		Invitrogen
BL21(DE3)pLysS			Protein expression strain		Novagen
pGEX-6P-1			GST fusion vector		GE Healthcare
pGEX*slr*			For expression of GST-Slr fusion protein		This study
pFW13		kan^R^	Streptococcal suicide vector		[52]
pMB01		kan^R^	*slr* knockout plasmid construct		This study
pCR®2.1-TOPO®		kan^R^, amp^R^	Cloning vector		Invitrogen

1 Presence of the *slr* gene was determined using PCR as described in Material and Methods.

2 WHO Collaborating Center for Reference and Research on Streptococci, Institute of Hygiene and Epidemiology, Prague, Czech Republic.

3 National Center for Disease Control reference codes.

### Generation of Slr mutant strain

A PCR product was generated from AP1 chromosomal DNA using oligonucleotide primers 5′-ATT-ATT-CTC-GAG-GGT-CTA-TTG-TTA-TCA-T-3′ and 5′-TTG-ACT-AGA-TCT-TAT-AAT-GGA-AGT-G-3′ with *Xho*I and *Bgl*II sites. The *Xho*I and *Bgl*II digested PCR fragment was ligated into *Xho*I and *Bgl*II digested pFW13 and transformed into TOP10 chemically competent cells (Invitrogen, Carlsbad, CA). Purified plasmids were electroporated into competent AP1 cells as previously described [24]. For preparation of competent AP1 cells, the bacteria were cultured over night in THY broth, washed with water and 0.5 M sucrose solution. The electroporator settings were 2.3 kV, 3 µF, 800 Ohms. The electroporated cells underwent phenotypic expression for 2 h in SOGAS solution at 37°C in 5% CO_2_. The AP1 recombinants were selected on THY-plates containing 150 µg/ml kanamycin. All the *S. pyogenes* isolates described in Table 1 were tested for the presence of the *slr* gene using standard PCR. DNA was prepared by boiling single colonies in 100 µl of distilled water followed by centrifugation at 10,000×*g* for 5 min. One µl of supernatant was used as template for PCR with an annealing temperature of 53°C using the oligonucleotide primers 5′-GCT-CCA-ACC-CCA-TTC-CC-ATC-3′ and 5′-TCG-TGA-GCA-TGA-CCT-TCT-TCA-TC-3′ generating an 1169 bp product.

### Characterization of mutants by Western Blot

The *S. pyogenes* wild type strain AP1 and mutants MB1 and MC25 were cultured in THY broth, including 150 µg/ml kanamycin for growth of the mutants, at 37°C in 5% CO_2_ atmosphere. The bacterial pellets were resuspended in SDS-PAGE sample buffer and separated by 10% SDS-PAGE and blotted to an Immobilion-P™ PVDF-membrane (Millipore, Bedford, MA) according to Towbin *et al* [25]. After blotting, the membrane was blocked in 15 ml PBS (140 mM NaCl, 30 mM KCl, 8 mM Na_2_HPO_4_, 2 mM KH_2_PO_4_) containing 0.1% Tween 20 (PBST) and 5% skim milk for 30 min at room temperature. After addition of primary antibody from Slr and M1 whole rabbit antiserum at dilution 1∶1,000 and 1∶20,000, the membrane was incubated at 37°C for 30 min in PBST and 5% skim milk. The filter was washed three times for 5 min in PBST and incubated with goat anti-rabbit IgG-peroxidase labeled antibody diluted 1∶3,000 in PBST and 5% skim milk. After incubation the filter was washed three times in PBST and developed with Supersignal® West Pico Chemiluminescent substrate (Thermo Scientific, Rockford, IL) followed by visualization using a Chemidoc XRS imaging system and Quantity One image analysis software (Bio-Rad).

### Analysis of bacterial mutants and Slr by electron microscopy

F(ab′)_2_ fragments of rabbit anti-Slr and anti-M1 IgG were generated using the IgG specific streptococcal protease IdeS [26]. IgG from 2 ml Slr or M1 antiserum was adsorbed to 2 ml Protein G Sepharose followed by washing with 8×5 ml PBS. 1 mg of GST-IdeS in 1 ml PBS was added and incubated for 5 h in 20°C. Filtrate was passed 5 times through Glutathione Sepharose (GE Healthcare, Uppsala, Sweden) to remove GST-IdeS. Integrity and purity of F(ab′)_2_ fragments were verified by SDS-PAGE and protein concentration was determined using Advanced Protein Assay Reagent (Cytoskeleton, Denver, CO) (data not shown). The presence and location of individual Slr and M1 molecules on wild type and mutant GAS was analyzed by negative staining and transmission electron microscopy as described previously [27]. F(ab′)_2_ fragment samples were conjugated with, 15 nm for Slr and 10 nm for M1, colloidal thiocyanate gold [28]. Bacteria were grown to Log phase (OD_620_ = 0.5), washed in 3×10 ml TBS (50 mM Tris-HCl, 150 mM NaCl, pH 7.4) and adjusted to 2×10^9^ colony forming units (cfu) per ml. The bacteria were mixed with the F(ab′)_2_ -Au conjugates and incubated for 1 h at room temperature at a 1∶2 molar ratio. 5 µl aliquots were adsorbed onto carbon-coated grids for 1 min, washed with two drops of water, and stained on two drops of 0.75% uranyl formate. The grids were rendered hydrophilic by glow discharge at low pressure in air. Specimens were observed in a JEOL JEM 1230 electron microscope operated at 80 kV accelerating voltage, and images were recorded with a Gatan Multiscan 791 CCD camera. A 5 µl aliquot of Slr (1 mg/ml) in TBS buffer (50 mM Triss-HCl, 150 mM NaCl, pH 7.4) was adsorbed onto carbon-coated grids and visualized by electron microscopy as previously described.

### Expression pattern of Slr and M1 protein

The *S. pyogenes* wild type strain AP1 was cultured in THY broth, at 37°C in 5% CO_2_ atmosphere. Growth was recorded hourly by measurement of OD_620_ and plotted in KaleidaGraph (Synergy Software, Reading, PA). Samples were collected hourly; Slr and M1 Western blot on cell extracts was performed as described above.

### Recombinant expression of Slr and purification of M1 protein

Slr was expressed in *Escherichia coli* using the GST Gene Fusion System (GE Healthcare). A 2337 base pair *slr* PCR product was amplified from *S. pyogenes* AP1 genomic DNA using primers 5′-ACT-TTG-GGA-TCC-TGT-CAA-TCA-CGA-3′ with a *Bam*HI site (underlined) and 5′-TCT-TAG-CTC-GAG-TTA-GTC-AGC-ATG-3′ with an *Xho*I site (underlined). This fragment was digested with *Bam*HI and *Xho*I, ligated into pGEX-6P-1 generating plasmid pGEX*slr*, that was used to transform *E. coli* BL21(DE3)pLys. pGEX*slr*/BL21(DE3)pLys was induced with 0.1 mM isopropyl β-D-thiogalactopyranoside and GST-Slr was purified using Glutathione-Sepharose (GE Healthcare) according to manufacturer's instructions. Purified GST-Slr was used for immunization of rabbits according to standard protocols [29]. The GST tag was removed from GST-Slr by on-column cleavage using Precission protease according to manufacturer's instructions (GE Healthcare). M1 protein was purified from culture supernatants from MC25 using affinity chromatography and anti-M1 rabbit antiserum was generated as previously described [23], [29].

### Sequence analysis and homology modeling of Slr

A homology model of Slr was generated using Multiple Mapping Method with Multiple Templates (M4T) (http://www.fiserlab.org/servers/m4t) [30] (using Internalin A (PDB 1O6V) [31] as a template. The Slr model was visualized using VMD 1.8.6 (http://www.ks.uiuc.edu/Research/vmd/) [32] and high resolution images were generated using the Tachyon ray tracer [33].

### Lipoprotein analysis using globomycin

AP1 bacteria were cultured in TH over night supplemented with 0, 12.5, 25 and 50 µg/ml of the signal peptidase II inhibitor globomycin similarly to what has been described for *S. equisimilis, S. equi* and *S. zooepidemicus* [34], [35]. Bacteria from 5 ml cultures were pelleted by centrifugation and resuspended in 200 µl PBS. Bacteria were lysed by addition of 100 U of the bacteriophage lysin PlyC [36] for 10 min at room temperature followed by addition of a final concentration of 25 µg/ml of ribonuclease A and deoxiribonuclease (Sigma) and incubation for 10 min at room temperature. Samples were centrifuged for 10 min at 14,000×*g* at 4°C. Pellets were resuspended in SDS-PAGE sample buffer and separated on 10% SDS-PAGE followed by transfer to PVDF membranes and anti-Slr Western blot as described above.

### Absorption of collagen type I to S. pyogenes strains

The strains AP1, MB1 and MC25 were grown in THY to OD_620_ = 0.8, with 150 µg/ml kanamycin for growth of the mutants, at 37°C in 5% CO_2_ atmosphere. The bacteria were washed with PBST and diluted to 2×10^9^ cfu/ml solution. 200 µl of the bacterial solution were incubated with 100 µg collagen I for one hour at room temperature. Aliquots of the solution were taken for electron microscopy visualization as previously described in Bober et al [27]. The bacterial solution was washed three times with PBST and the bound collagen was eluted from bacterial surface by incubation with 0.1 M glycine, pH 2.0 for 20 min. The bacteria were removed by centrifugation, 1500×*g* for 10 minutes, and the supernatant was neutralized by addition of 1 M Tris base. 15, 10 and 5 µl of the samples were immobilized on a PVDF membrane using the Milliblot-D system (Millipore). The membrane was blocked in 5 ml PBST and 5% skim milk for 30 min at room temperature. After addition of rabbit anti-collagen I/III antibody (Calbiochem, Darmstadt, Germany) diluted 1∶60 in PBST and 5% skim milk, the membrane was incubated at 37°C for 30 min. The filter was washed three times for 5 min in PBST and incubated with goat anti-rabbit IgG-peroxidase labeled antibody (Bio-Rad) diluted 1∶3,000 in PBST and 5% skim milk. After incubation, the filter was washed three times in PBST and visualized as above. The samples were also analyzed by separation on a reducing 3–12% SDS-PAGE gel and stained with Coomassie Brilliant Blue.

### Radiolabeling of collagen type I and Slr

Radioactive iodine (^125^I) labeling was performed using IODO-BEADS (Pierce, Rockford, IL, USA) and done according to the manufacturer's instructions. Unbound iodine was removed on a PD-10 column (Amersham Biosciences, Uppsala, Sweden) in PBS buffer containing 0.05% Tween 20 and 0.002% sodium acide (PBSAT). Fractions of 500 µl were collected and radioactivity was measured using a Wallac Wizard 1470 automatic gamma counter. The fraction containing the radiolabeled protein was stored at +4°C until further usage.

### In vitro binding studies

The strains AP1, MB1 and MC25 were grown in THY to OD_620_ = 0.8, with 150 µg/ml kanamycin for growth of the mutants, at 37°C in 5% CO_2_ atmosphere. The bacteria were washed with PBSAT and diluted to 2×10^9^ cfu/ml solution. 250 µl of the bacterial solution were incubated with approximately 100 0000 counts per minute (cpm) ^125^I labeled collagen I for 30 min at room temperature. The unbound collagen was removed by centrifugation, 1500×*g* for 10 minutes, and the pellet was measured for radioactivity using a Wallac Wizard 1470 automatic gamma counter. Percent of binding was calculated with KaleidaGraph (Synergy Software) and values were illustrated in a dot plot. Experiments were performed in quadruples at three independent time points.

Collagen I, non-denatured and denatured with guanidine hydrochloride was applied directly onto a PVDF membrane using the Milliblot-D system (Millipore). The membrane was washed twice for 30 min in PBS containing 0.25% Tween 20 and 2% bovine serum albumin, incubated with ^125^I-Slr overnight at 4°C, and washed three times for 20 min in blocking buffer as described above. Signals of bound ligand were analyzed by phosphoimaging in a Fujix BAS 2000 Bioimaging analyzer (Fujifilm Sverige AB, Stockholm, Sweden).

The presence and location of individual Slr molecules on monomeric and fibrillar collagen I was analyzed by gold labeling, negative staining and transmission electron microscopy as described above.

### Surface plasmon resonance (SPR) interaction analysis

Collagen I, diluted to 10 mg/liter in 10 mM sodium acetate (pH 4), was immobilized via amine coupling to CM3 sensorchip flow chambers (GE Healthcare) at moderate response levels (e.g. 1500 RU). Briefly, Collagen was mixed with freshly prepared 100 mM N-hydroxysuccinimide and 400 mM N-ethyl-N'-(dimethylaminopropyl)carbodiimide in equal volumes, and capping of unreacted carboxymethyl sites was achieved by a 1 M ethanolamine (pH 8) injection. A flow chamber subjected to the immobilization protocol but without any addition of protein was used as control (blank) for each experiment. Slr and M1 proteins respectively, were sequentially diluted 2-fold in running buffer (10 mM HEPES, 150 mM NaCl, and 0.005% Surfactant P20 (GE Healthcare) pH 7.5) and injected over the collagen surface starting at 500 nM and 35 µl/min. Bacterial cell suspensions were diluted 2-fold into PBS for injections in PBS in sequence starting from 2×10^9^ cfu/ml solution. Binding was monitored in a BIAcore 2000 instrument. Between experiments, the collagen surfaces were strictly regenerated with multiple pulses of 2 M NaCl and 1.5 M glycine-HCl pH 2.0 followed by an extensive wash procedure using running buffer or PBS, respectively. After x-axis and y-axis normalization of obtained data, the blank bulk refraction curves from the control flow chamber of each injected concentration or experiment were subtracted. Binding curves were displayed and where applicable, the association (K_a_) and dissociation (K_d_) rate constants were determined using the BIA Evaluation 4.1 software and its equation for 1∶1 Langmuir binding.

## Results

### Slr is a horseshoe shaped LRR lipoprotein

**Figure 1:**
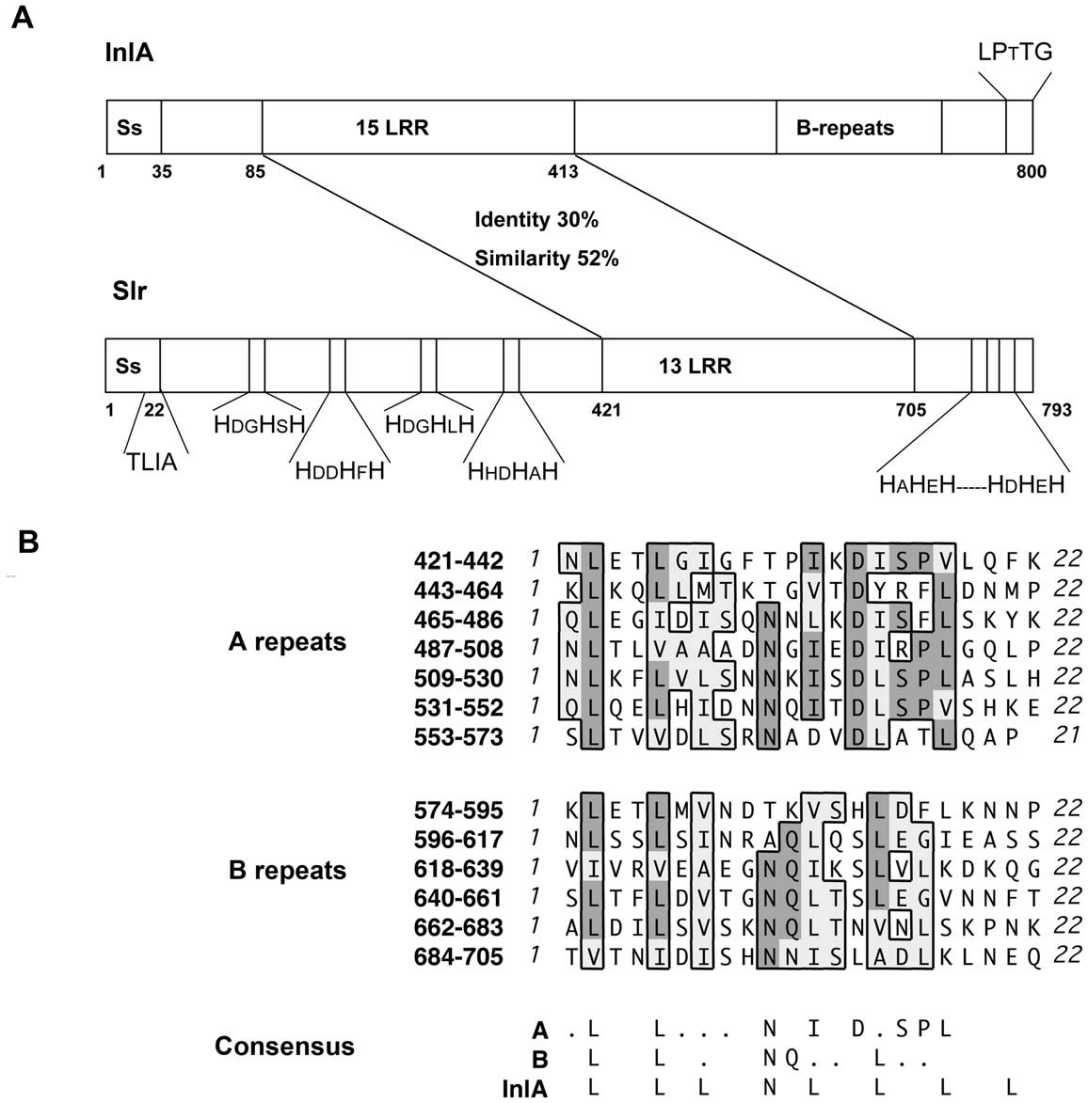
Characteristics of the Slr protein. A: Schematic comparison of the InlA from *L. monocytogenes* and Slr. The identity and similarity between the two LRR regions are stated. The cell wall anchoring motif for InlA and the signal peptide II recognition motif for Slr are indicated as well as the histidine triad motifs. B: Sequence alignments of the two groups of LRRs (A and B repeats) in Slr and comparison of the LRR consensus sequences of Slr and InlA.

**Figure 2:**
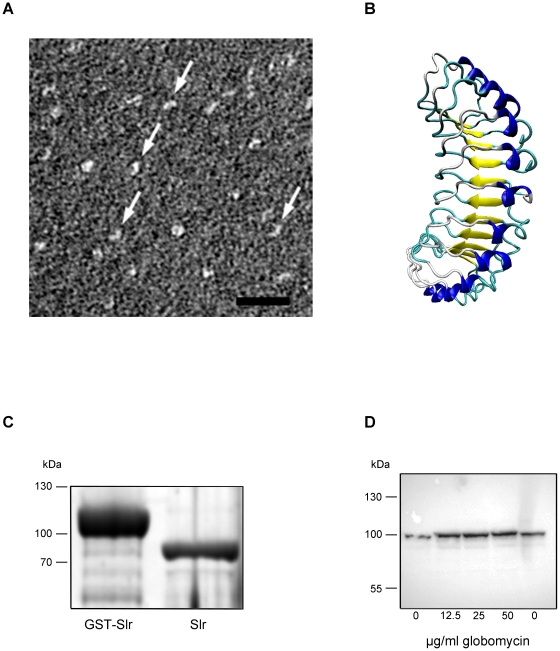
Visualization and confirmation of the LRR lipoprotein Slr. A: Slr protein visualized by electron microscopy showing the typical horseshoe shape of a LRR protein. Scale bar  = 25 nm. B: a homology model of Slr using InlA as the template. The 11 LRRs forming β-sheets in yellow, α−helices in blue, and loops in turquoise. C: SDS-PAGE analysis of recombinantly expressed GST-Slr and Slr with the GST tag cleaved off. D: Western blot analysis, using anti-Slr antibodies, of bacterial cell extracts from AP1 bacteria grown in the presence of increasing amounts of the signal peptidase II inhibitor globomycin as indicated.

The *slr* gene encoding the *S. pyogenes* LRR protein Slr is present in 32/32 strains of 24 different M serotypes as determined by PCR (Table 1) and is also present in all currently sequenced *S. pyogenes* genomes. The *slr* gene in strain AP1 was cloned and sequenced using oligonucleotide primers based on the genome sequence from the M1 strain SF370 (Accession no. NC_002737) [37]. Sequencing of this gene revealed an ORF encoding a 793 amino acid protein that is 99% identical to the predicted Slr protein from SF370. The sequences of the Slr from AP1 and the corresponding gene *slr*, have been deposited in Genbank under the accession no. HQ908654. Slr contains a 21 amino acid putative N-terminal signal sequence ending with the amino acid sequence TLIA. This is the recognition sequence for signal peptidase II that allows for lipid modification of the resulting amino-terminal cysteine and insertion into the cellular membrane. In between amino acid 22 and 421 are four histidine triad motifs, that are not present in InlA of *L. monocytogenes*. There are 13 LRRs in Slr spanning over amino acid numbers 421–705 forming the β-sheets compared to 15 LRRs in InlA. The carboxyl terminal end of Slr contains histidine rich repeat sequences, but lacks a cell wall anchoring motif, while InlA carries a classical LPtTG cell wall anchoring motif (Figure 1A). The LRR in Slr resembles the consensus motif of the LRR region in InlA (Figure 1B). To confirm the horseshoe shape of the protein, Slr was visualized by electron microscopy. The Slr is pointed out by arrows and the shape is well visible supporting a horseshoe like conformation of Slr (Figure 2A). Homology modeling of Slr using InlA as the template further supported the protein's structure. The modeled LRRs forming the β-sheets (blue) are suggested to be the protein-protein interaction sites (Figure 2B).

### Slr is a surface lipoprotein

**Figure 3:**
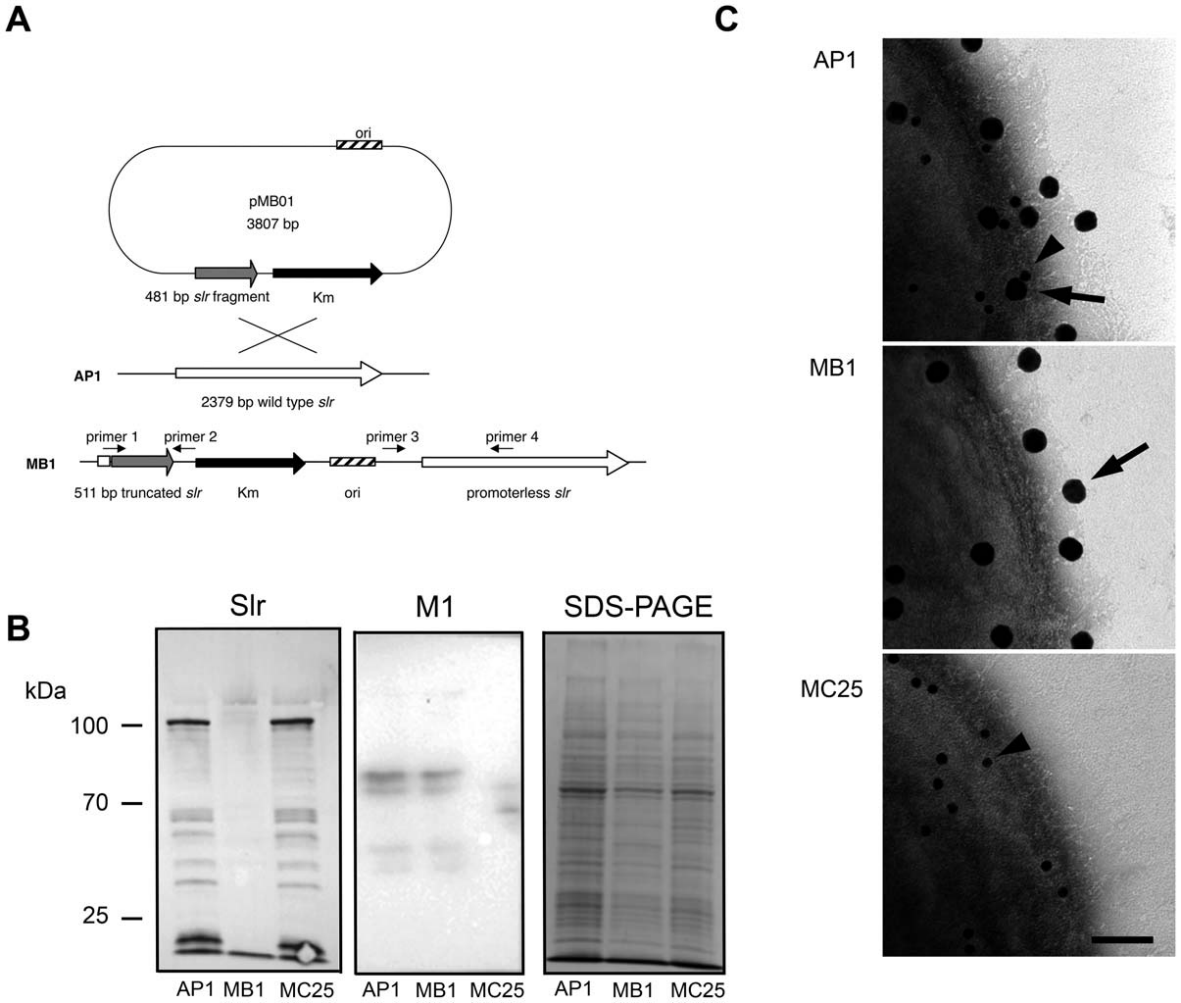
Slr gene disruption, mutant strain analysis and protein localization by electron microscopy. A: Schematic representation of the mutagenesis strategy used to disrupt *slr* in *S. pyogenes* strain AP1. B: SDS-PAGE and Western blot analysis, using antibodies against Slr and M1, of bacterial cell extracts of the mutant strains MB1 and MC25, and wild type bacteria AP1. C: Wild type strain AP1, mutant strain MB1 and mutant strain MC25 incubated with gold labeled F(ab')_2_ fragments of anti-Slr (small gold particles) and anti-M1 (big gold particles) IgG. The dots represent binding of the F(ab')_2_ fragments to the Slr and M1 protein on the bacterial surface. Arrowheads without arrows indicate binding of anti-Slr antibodies, and complete arrows indicate binding of anti-M1 antibodies in all three panels. Scale bar  = 100 nm.

**Figure 4:**
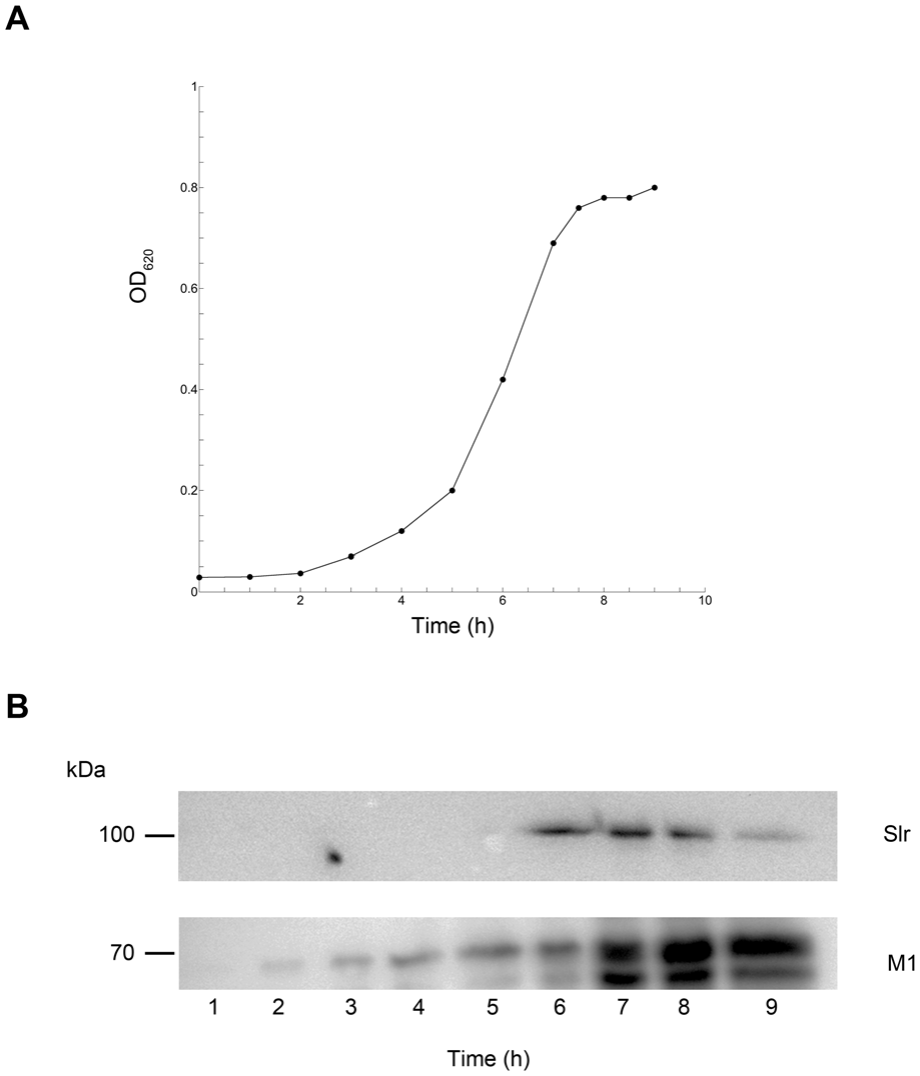
Growth curve and expression pattern of Slr and M1 protein in *S. pyogenes* strain AP1. A: Growth curve of *S. pyogenes* wild type strain AP1 growth in THY medium over time. B: Western blot analysis of Slr and M1 protein expression in strain AP1 over 9 hours of growth.

The Slr protein was expressed in *E. coli* using the GST Gene Fusion system and was used in several *in vitro* experiments (See Material and Methods). GST-Slr and Slr were separated on a SDS-PAGE for size and purity control. The GST-Slr has an apparent size of 110 kDa and Slr of approximately 85 kDa (Figure 2C). To confirm that Slr is a lipoprotein, a globomycin inhibition assay was performed. The maturation of lipoproteins is governed by signal peptidase II that removes signal peptides in the NH_2_-terminal signal sequence. This step is inhibited by globomycin, resulting in a surplus of signal peptides and thus an increased molecular size (Figure 2D), confirming that Slr is a lipoprotein. To investigate the role of Slr on the surface of *S. pyogenes*, we constructed a Slr mutant by single crossover mutagenesis of the AP1 strain of M1 serotype. A 505 bp promotorless fragment of the *slr* gene was cloned into multiple cloning site I of pFW13, resulting in vector pMB01 (Figure 3A). One mutant strain denoted MB1 was chosen for further analysis. Correct insertion of pMB01 was confirmed by PCR using gene- and plasmid specific primers (Figure 3A, primer 1–4, data not shown). To confirm *slr* disruption in MB1 and the Slr and M1 expression in AP1 and MC25, protein extracts from AP1, MB1 and MC25 cell pellets were analyzed by immunoblotting using anti-Slr and anti-M1 antibodies. A strong signal was detected in the AP1 pellet for both Slr and M1 protein. The only detectable signal in MB1 was for the M1 protein while in MC25 only Slr expression was detected (Figure 3B). Further mutant strain analysis and surface protein localization was done using gold labeled F(ab')_2_ fragments of anti-Slr (small gold) and anti-M1 (large gold) IgG. The F(ab')_2_ fragments were incubated with wild type and mutant strains and analyzed by negative staining and transmission electron microscopy. The AP1 strain, expressing both Slr and M1 protein, bound both M1 and Slr antibodies simultaneously, (Figure 3C) whereas the mutant MB1 strain only showed binding of M1 antibodies verifying that the Slr protein is not expressed on the surface (Figure 3C). The mutant MC25 strain only exhibited binding of Slr antibodies, showing that the M1 protein is not present on the bacterial surface (Figure 3C). The expression pattern of Slr and M1 was investigated in the wild type strain AP1. The growth was plotted as OD_620_ versus the incubation time (Figure 4A), and samples were taken for Western blot analysis. Slr protein can be detected from hour 6 (approx. OD_620_ = 0.4) and declines at hour 9 (approx. OD_620_ = 0.8), while M1 can be detected from hour 2 (approx. OD_620_ = 0.05) (Figure 4B) and starts to be degraded at hour 7 (data not shown). Taken together, these results indicate that Slr is an abundant membrane bound lipoprotein that is co-expressed on the surface with the M1 protein in early stationary growth phase.

### Slr and M1 binds to collagen type I

**Figure 5:**
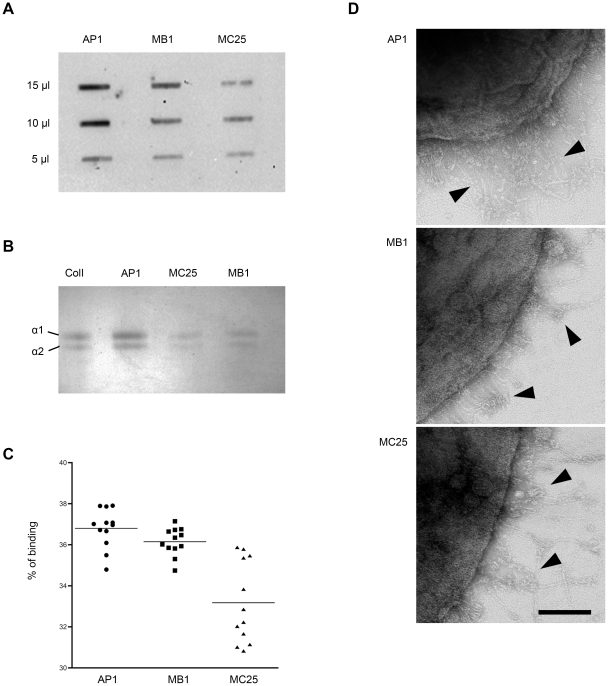
Absorption of collagen type I to *S. pyogenes* strains. A: After incubation of mutant strains MB1, MC25 and wild type AP1 strain with collagen I, low pH eluates from bacteria were immobilized on a PVDF membrane and detected using anti-collagen antibodies. The amount of collagen I eluate applied on the membrane is indicated to the left. B: The bacterial eluates described in 4A were separated on SDS-PAGE and stained with Coomassie Brilliant Blue. Visible are the α1 (134 kDa) and α2 (130 kDa) chains indicated with arrows. C: Binding in percentage of ^125^I labeled collagen I to the bacterial strains AP1, MB1 and MC25. Experiments were performed in quadruples at three independent time points. D: Interaction between *S. pyogenes* strains and monomeric collagen. Collagen I was incubated with AP1, MB1 and MC25 and visualized by electron microscopy. Arrows point to collagen I monomers. Scale bar  = 100 nm.

**Figure 6:**
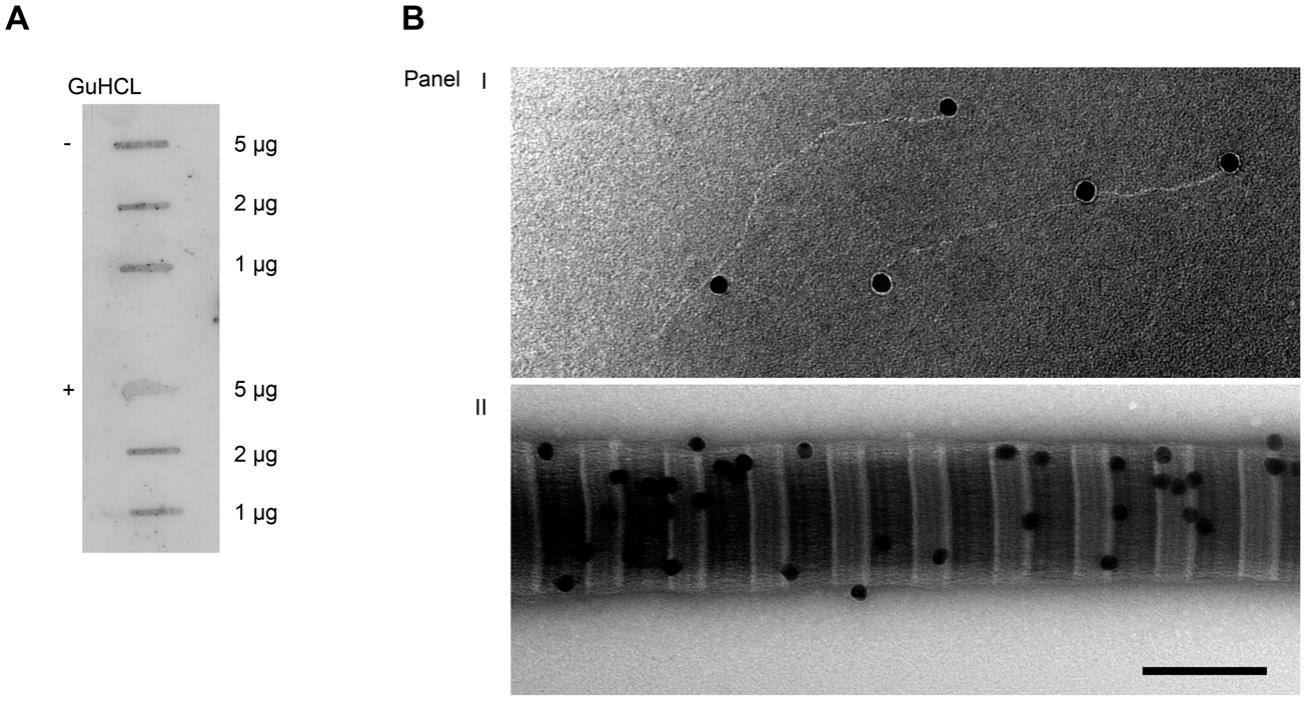
Binding of Slr to immobilized collagen I. A: Collagen I denatured with 4 M guanidine hydrochloride (+) and non-denatured collagen I (−) were applied to a PVDF membrane and incubated with radiolabeled Slr. The amount of collagen is indicated to the right. B: Binding of gold labeled Slr (the dots) and collagen I using electron microscopy. The dots are bound to C- and N terminal as well as 70 respectively 100 nm further in on the monomeric collagen I (panel I). The short span between light strands represents the overlap region, whiles the dark span represents the gap region on a collagen I fibril. Binding can be observed in both regions, but is mostly concentrated to the overlap region (panel II). Scale bar  = 100 nm.

**Figure 7:**
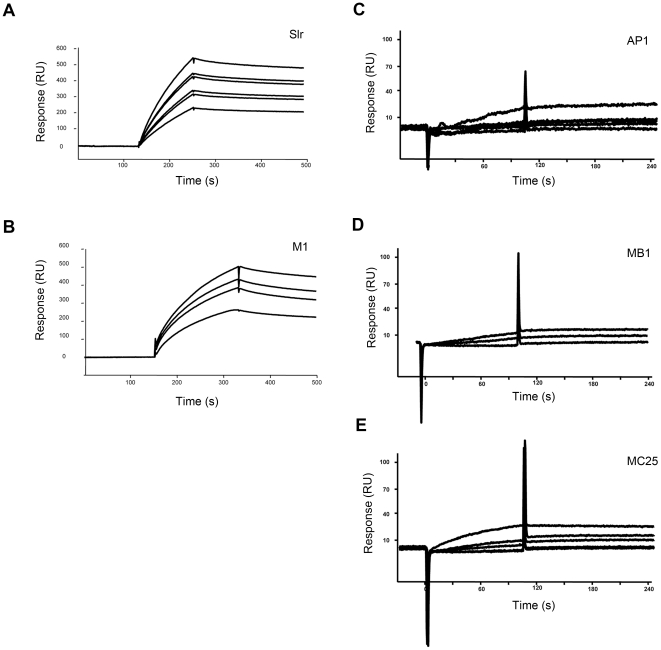
Surface plasmon resonance interaction analysis. The binding of Slr and M1 protein, the wild type strain AP1, and mutant strains MB1 and MC25 strains to immobilized collagen I was investigated using surface plasmon resonance. Binding curves are displayed and, the association (K_a_) and dissociation (K_d_) rate constants were determined. A–B: Slr and M1 proteins were diluted 2-fold in running buffer and injected over the collagen surface starting at 500 nM and 35 µl/min. The proteins displayed a dose-dependent binding to collagen I with a K_D_ of 12 nM for Slr and 54 nM for M1 protein. C–E: Bacterial cell suspensions were diluted 2-fold into PBS for injections sequences starting from 2×10^9^ cfu/ml solution.

Previous results implicated that M proteins can interact with collagens (data not shown). Since it has been stipulated that LRRs have collagen binding capacity and since Slr is co-expressed with M protein on the bacterial surface there are reasons to investigate whether Slr can contribute to the collagen binding capacity of *S. pyogenes*. Therefore, we analyzed a possible Slr- collagen interaction. Wild type AP1 (expressing both M protein and Slr), mutant strain MB1 (lacking Slr protein), and MC25 (lacking M1 protein) bacteria were incubated with collagen I and the bound collagen was eluted with low pH glycine. The eluted proteins were immobilized on a PDVF membrane and anti-collagen Western blot was performed. The wild type AP1 strains exhibited strongest binding ability towards collagen I, while MB1 and MC25 strain showed diminished binding capacity (Figure 5A). The eluted collagen I from the AP1, MB1 and MC25 strains was also separated on 3–12% SDS-PAGE with collagen I as a standard and similar results could be observed as for anti-collagen Western blot, with AP1 showing a higher binding of collagen I than both MC25 and MB1 (Figure 5B). In order to further establish the collagen binding capacity of strains AP1, MB1 and MC25, a binding assay with radiolabeled collagen I was performed. The strains were incubated with ^125^I labeled collagen I, unbound collagen was removed by centrifugation and the radioactivity value of bound collagen was measured. All strains exhibited binding toward collagen I with the binding capacity of 36.8% for AP1, 36.1% for MB1 and 33.2% for MC25 (Figure 5C). The binding of collagen to the bacterial strains was also visualized by electron microscopy, where association of collagen I could be observed for all three strains with a tendency of less accumulation in MB1 and MC25 (Figure 5D). To determine the influence of collagen denaturation during SDS-PAGE on the Slr binding, non-denatured and guanidine hydrochloride-denatured collagen I was examined in a slot blot experiment. The same procedure was used to determine that the binding was not caused by the GST-tag on Slr. The slot blot experiment showed no interaction between collagen I and the GST-tag (data not shown). The radiolabeled Slr bound to both collagen preparations in a similar manner (Figure 6A). Further *in vitro* interaction between Slr and both monomer and fibrillar collagen I was investigated. Gold labeled Slr (the dots) and monomer collagen I show four binding sites. The dots are bound to C- and N terminal as well as 70 respectively 100 nm further in on the collagen (Figure 6B, panel I). More detailed analysis is required to identify the specific site of interaction. The collagen I fibril is composed of triple helices overlapping each other, resulting in a variation of triple helical thickness in the molecule. The short span between light strands represents the overlap region where the collagen triple helices overlap each other, while the dark span represents the gap region where the gap between collagen helices occurs. Binding can be observed in both regions, but is mostly concentrated to the overlap region (Figure 6B, panel II). These experiments were also executed with gold-labeled GST. There was no interactions between GST and monomeric or fibrillar collagen I (data not shown). The binding of Slr and M1 protein as well as the AP1, MB1 and MC25 strains to collagen I was investigated using surface plasmon resonance. Slr and M1 showed a dose-dependent binding to collagen I with a K_D_ of 12 nM and 54 nM, respectively (Figure 7A and B). The bacterial strains AP1, MB1 and MC25 also showed a dose-dependent binding to collagen I (Figure 7C–E). Taken together, these results suggest that both Slr and M1 protein can bind collagen I, both when in purified form and in a bacterial surface context. Slr has a somewhat higher affinity towards collagen I than M1.

## Discussion

*S. pyogenes* is a human pathogen responsible for a variety of diseases, from common infections to life-threatening conditions. One of the most studied virulence factors of *S. pyogenes* is the cell wall anchored M protein. It is involved in adherence to human epithelial cells, such as buccal cells and epidermal keratinocytes [38], [39]. The M protein is also intimately linked to antiphagocytic properties and intracellular survival in phagocytic cells [40], [41]. Of particular interest for the present study is that the M6 protein present on the surface of *S. pyogenes* of M6 serotype has been suggested to camouflage the LRR lipoprotein Slr from Slr antibody recognition [19].

Lipoproteins from Gram-positive bacteria are not as well studied as cell wall anchored proteins, but several recent studies have suggested that lipoproteins also are important for immune evasion and adherence during colonization and infection. Furthermore, several lipoproteins have shown promise as vaccine candidates [42]. Previously described lipoproteins of *S. pyogenes* are linked to metal transport as acquisition, most of them belong to ABC transport systems and some have been shown to be virulence factors in animal models of infection [43], [44], [45], [46].

Proteins containing LRRs vary widely in functions and are found in both prokaryotes and eukaryotes, but the known bacterial LRR proteins, especially from *Listeria* species, are important virulence factors that mediate adhesion and cellular invasion through protein-protein interactions [17]. The *slr* gene encoding Slr can be found in all 32 strains of 23 different M serotypes *S. pyogenes* used in this study, as well as in all currently sequenced *S. pyogenes* genomes. The apparent conservation of the *slr* gene, the previous finding that patients with *S. pyogenes* infections seroconvert to the Slr protein and the fact that Slr negative mutants are attenuated in animal model and contribute to phagocytosis resistance [18], warrant further investigation of the function of Slr and possibly also exploration of Slr as a vaccine candidate.

Slr and M1 protein expression on the bacterial surface reach their peaks at different time points during the logarithmic growth phase and M1 starts to be degraded before Slr. However, during a substantial amount of time in late logarithmic growth phase, both proteins are present at the bacterial surface. This expression pattern could be similar in an *in vivo* situation, where Slr and M1 surface exposure results in optimal adherence to host tissue. Direct binding experiments indicate that Slr binds strongly to collagen I, both in native and denatured form. This could suggest a fairly limited site of interaction and/or a protein-protein interaction that is not dependent on the three-dimensional structure of collagen I. *In vitro* binding assay with the wild type and mutant strains showed that elimination of either Slr or the M1 protein on the bacterial surface did not have a major affect on the strains' total capacity to bind collagen I. A similar binding pattern of Slr and M1 toward collagen could also be observed in direct binding assays, leading to a conclusion that both proteins can use collagen as a ligand.

Interestingly, we did not observe that M1 protein inhibited the binding of gold-labeled Slr antibodies to Slr on the bacterial surface as previously described for the M6 protein [19]. In fact, gold-labeled anti-M1 and anti-Slr antibodies could bind simultaneously to the bacterial surface (Fig. 3C). This observation, in combination with observed collagen binding to both proteins on the surface of the bacteria, suggest that under the conditions studied here, M1 and Slr are simultaneously accessible for some protein-protein interactions on the bacterial surface. On the other hand, elimination of either Slr or the M1 protein from the bacterial surface might reveal additional binding sites toward collagen I on the remaining protein in concordance with the camouflaging described by Waldemarsson *et al* [19]. This might explain why there was not a major reduction in total collagen I binding capacity of the Slr and M1 mutants compared to the wild type strain.

Another feature of Slr are the four histidine triad motifs in the N-terminal part of the molecule, which are absent in InlA of *L. monocytogenes*. Such motifs have been identified in four proteins of *Streptococccus pneumoniae* and might be involved in metal or nucleoside binding [47]. Furthermore, such histidine triad proteins in *S. pneumoniae* have been shown to alter complement deposition of the bacterial surface [48]. Therefore, enzymatic or other functions than collagen binding of Slr, cannot be excluded.

In conclusion, we have established that Slr is an abundant membrane bound horseshoe shaped LRR lipoprotein from *S. pyogenes* that, both alone and in concert with M1 protein, interacts strongly with collagen I. The multiple interactions with collagen I through Slr and M protein, indicate that collagen binding is an important feature of *S. pyogenes*. Therefore, further studies elucidating the regulation of, and possible inhibition/co-operation between Slr and M proteins will be important in understanding how *S. pyogenes* interacts with the extracellular matrix during colonization and infection.
